# Editorial: Emotional functioning and post-traumatic outcomes in the aftermath of a traumatic event

**DOI:** 10.3389/fpsyg.2022.1116690

**Published:** 2022-12-23

**Authors:** Annunziata Romeo, Lorys Castelli, Marialaura Di Tella

**Affiliations:** Department of Psychology, University of Turin, Turin, Italy

**Keywords:** emotional functioning, trauma, post-traumatic growth (PTG), post-traumatic stress, anxiety/depressive symptoms

The terrifying aftermath of trauma can cause both negative and positive psychological outcomes. Traumatic events can induce people to experience a series of strong negative emotional responses, resulting in symptoms of psychological distress, such as post-traumatic stress symptoms (PTSS) and anxiety/depressive symptoms (e.g., Wang et al., 2005). Conversely, positive outcomes usually identify with post-traumatic growth (PTG), which refers to the experience of positive psychological change that occurs as a result of the struggle with highly challenging life circumstances (Tedeschi and Calhoun, 2004).

In the occurrence of both negative and positive psychological reactions, a series of factors can play an important role (e.g., emotional functioning and coping strategies) (Romeo et al., 2019, 2022).

The present Research Topic aimed to shed further light on controversial aspects concerning the psychological consequences of a traumatic event, collecting contributions from different countries and fields.

Overall, we have twelve accepted papers. Half of these papers reported results of post-traumatic outcomes associated with the COVID-19 outbreak. Particularly, Lamiani et al. conducted a grounded theory based on the experience of 24 clinical psychologists who provided extensive support to the population during the pandemic in Italy. Results of the focus groups showed that repositioning (i.e., dealing with and integrating unpleasant emotional experiences deriving from the pandemic through different coping strategies) was the core task people had to face after the emergency phase of COVID-19.

Healthcare workers were also the focus of the study by Zakeri et al. The authors investigated mental health in nurses before and during the first wave of the COVID-19 pandemic in Iran. Results showed that while the level of burnout remained the same, anxiety, stress and depression increased significantly during the COVID-19 pandemic.

Another study of Zakeri et al. aimed to compare the compassion satisfaction, compassion fatigue and hardiness among nurses before and during the COVID-19 outbreak.

Although no significant differences were found between these psychological aspects before and during the COVID-19 outbreak, results showed that hardiness was a significant predictor of both compassion satisfaction and compassion fatigue.

The traumatic impact of COVID-19 on healthcare workers' mental health has also been examined by Yeung et al. They found that higher COVID-19-specific worries, higher perceived stigma of being a healthcare worker, and lower work satisfaction predicted higher anxiety symptoms in nurses in Hong Kong.

The direct and indirect impact of the COVID-19 pandemic was examined by two studies. Particularly, Taurisano et al. investigated psychological outcomes in a group of patients infected with COVID-19 comparing them to a sample of healthy participants. Results showed significant gender differences, with women reporting higher scores than men, in PTSS, depressive symptoms and representation of interpersonal distance in the clinical group only. The study of Bhushan et al. aimed to examine the direct (death or hospitalization in the family) and the indirect (media reports of the COVID situation) exposure of COVID-19 experience on children and adolescents and its subsequent relationship with PTSS and PTG during the second wave of COVID-19 in India. Overall, results revealed that 68.9% of them had PTSS, and 39.8% of those reporting PTSS were also experiencing PTG. Both direct and indirect exposure of COVID-19 was associated with higher PTSS. Arousal appeared to be the most frequently reported traumatic symptom.

The other four accepted studies sought to investigate post-traumatic outcomes in different other traumatic events. Fausor et al. examined long-term PTG in adults directly exposed to terrorist attacks in Spain (time span: 2–47 years earlier). Results revealed gender differences in PTG levels, with women reporting higher scores than men, and a positive linear relationship between PTG and cumulative trauma after the terrorist attacks. Significant positive associations were also detected between some PTG dimensions (i.e., appreciation of life and spiritual change) and PTSS.

The study of Rowe et al. aimed to analyze mental health symptoms among first aiders exposed to different traumatic events. As expected, rates of mental health outcomes in first aiders were higher than in the general population. Particularly, women reported higher levels of PTSS than men, and a significant correlation between the number of traumatic events and years of experience was detected.

Ziȩba et al. examined the associations between prioritizing positivity, styles of rumination, coping strategies, and PTG in a group of participants exposed to different critical events. Two evaluations were conducted and a series of validated measures were administered. Results revealed that PTG was positively associated with prioritizing positivity, deliberate rumination, and religious coping, whereas negative relationships were found between the former and intrusive rumination.

Furthermore, Zeighami et al. conducted a qualitative study that aimed to investigate the effects of sexual harassment in the workplace on Iranian nurses. From the content analysis four subcategories have been extracted: “psychological trauma,” “detrimental effects of work,” “physical problems,” and “disintegration of warm family relationships.” In sum, sexual harassment had a greater negative psychological consequences for nurses and had a significant burden on the healthcare system due to decreased productivity and loss of active labor.

Finally, two papers examined the association between post-traumatic outcomes and cognitive and emotional abilities. Particularly, the study of Elam and Taku aimed to examine how perceived PTG and resiliency were, respectively, associated with empathy and emotion recognition in a group of college students. Results showed that perceived PTG and resilience were related to different cognitive abilities. In fact, PTG significantly predicted increased emotion recognition but not empathy, whereas resilience was found to be negatively associated with empathy but not with emotion recognition.

Mariani et al. explored whether emotional processes perform different functions during waking thoughts and night dreams during the first lockdown in Italy. Two different processes of emotional elaboration emerged: the use of greater symbolization processes during dreams and a higher emotional distance in waking thoughts. Findings suggested a greater contact with the processing of trauma during the nocturnal processes, and a greater use of defensive strategies during the diurnal processes.

In conclusion, the studies included in the present Research Topic have shown that both positive and negative post-traumatic outcomes may emerge in the aftermath of a traumatic event. Some studies have highlighted that PTSS and PTG can co-occur, with socio-demographic and psychological differences that seem to characterize the levels of positive and negative outcomes. Particularly, women have been found to experience higher levels of both growth and PTSS than men, whereas distinct emotional and cognitive processes have been shown to predict PTG and PTSS. On the one hand, deliberate rumination, religious coping and emotion recognition abilities seem to promote PTG, while, on the other hand, intrusive rumination and symbolization processes during dreams appear to predispose individuals to high levels of distress.

Given the complexity of those relationships, further research is needed to clarify the association between negative and positive psychological outcomes in the aftermath of a traumatic event.

## Author contributions

Conceptualization and writing—original draft preparation: MD and AR. Writing—review and editing: AR and LC. Supervision: MD. All authors have read and agreed to the published version of the manuscript.
